# How Electrical Heterogeneity Parameters of Ion-Exchange Membrane Surface Affect the Mass Transfer and Water Splitting Rate in Electrodialysis

**DOI:** 10.3390/ijms21030973

**Published:** 2020-02-01

**Authors:** Svetlana Zyryanova, Semyon Mareev, Violetta Gil, Elizaveta Korzhova, Natalia Pismenskaya, Veronika Sarapulova, Olesya Rybalkina, Evgeniy Boyko, Christian Larchet, Lasaad Dammak, Victor Nikonenko

**Affiliations:** 1 Department of Physical Chemistry, Kuban State University, 149 Stavropolskaya st., 350040 Krasnodar, Russia; zyryanova.s.v@yandex.ru (S.Z.); mareev-semyon@bk.ru (S.M.); violetta_gil@mail.ru (V.G.); n_pismen@mail.ru (N.P.); vsarapulova@gmail.com (V.S.); olesia93rus@mail.ru (O.R.); boykoes@yandex.ru (E.B.); 2 Institut UTINAM (UMR CNRS 6213), Université de Bourgogne-Franche-Comté, 16 Route de Gray, 25030 Besançon CEDEX, France; lizelotavocal@mail.ru; 3 Institut de Chimie et des Matériaux Paris-Est, UMR7182 CNRS–Université Paris-Est, 2 Rue Henri Dunant, 94320 Thiais, France; clarchet4@gmail.com (C.L.); dammak@u-pec.fr (L.D.)

**Keywords:** ion-exchange membrane, surface modification, electrodialysis, electroconvection, chronopotentiometry, voltammetry

## Abstract

Electrodialysis (ED) has been demonstrated as an effective membrane method for desalination, concentration, and separation. Electroconvection (EC) is a phenomenon which can essentially increase the mass transfer rate and reduce the undesirable water splitting effect. Efforts by a number of researchers are ongoing to create conditions for developing EC, in particular, through the formation of electrical heterogeneity on the membrane surface. We attempt, for the first time, to optimize the parameters of surface electrical heterogeneity for ion-exchange membranes used in a laboratory ED cell. Thirteen different patterns on the surface of two Neosepta anion-exchange membranes, AMX and AMX-Sb, were tested. Low-conductive fluoropolymer spots were formed on the membrane surface using the electrospinning technique. Spots in the form of squares, rectangles, and circles with different sizes and distances between them were applied. We found that the spots’ shape did not have a visible effect. The best effect, i.e., the maximum mass transfer rate and the minimum water splitting rate, was found when the spots’ size was close to that of the diffusion layer thickness, *δ* (about 250 μm in the experimental conditions), and the distance between the spots was slightly larger than *δ*, such that the fraction of the screened surface was about 20%.

## 1. Introduction

Today electrodialysis (ED) is a well-developed membrane technique which has many applications, such as desalination and concentration of solutions, separation of ions, acid and alkali production, energy generation, and others [1,2,3,4].

In the course of electrodialysis of dilute solutions, the surface properties of ion-exchange membranes (IEM) significantly affect the performance of the separation process. In particular, the membrane surface parameters have a strong impact on the development of the effects coupled with concentration polarization: first of all, on the intensity of electroconvection (EC) and generation of H^+^ and OH^–^ ions occurring at the membrane/solution interface [5,6,7,8,9,10,11,12].

The fact that the current density, *i*, in ion-exchange membrane systems can be higher than the limiting current density, *i_lim_* (attained when the electrolyte concentration becomes much lower than the concentration in the bulk solution), has been known for a long time [13]. The appearance of additional charge carriers (H^+^ and OH^–^ ions), formed as a result of water splitting in a membrane system [14], has long been considered as the main and sometimes the only reason for the current increase at *i* > *i_lim_* [15]. EC is the transfer of fluid under the action of an electric force applied to the space charge in solution. The most frequently discussed mechanism of EC in the literature is the electroosmotic slip occurring when an electric force is applied to the space charge in the depleted solution located at the membrane surface [16]. The generation of H^+^ and OH^–^ ions is, in most cases, an undesirable process during electrodialysis, leading to a decrease in current efficiency and a change in the pH of the solutions. The latter is associated with an increased risk of deposition of hardness salts [17,18]. In contrast to the effect of H^+^ and OH^–^ ion generation, EC is considered as an extremely desirable effect which not only causes a significant increase in mass transfer [8,10,19,20], but also leads to a decrease in the water splitting rate [17,18,21,22]. This effect is explained by the fact that an increase in EC mixing of the near-membrane solution layer leads to an increase in the concentration of salt ions above the critical value at which the generation of H^+^ and OH^–^ ions begins.

The properties of the IEM surface, which affect the EC intensity and the rate of H^+^ and OH^–^ ion generation, include the electrical and geometric heterogeneity of the surface [12], the degree of its hydrophobicity/hydrophilicity [12], and the surface charge [23]. The membrane bulk properties do not influence electroconvection. In this regard, intensification of EC, which can significantly improve the membrane performance, may be obtained by a targeted surface modification with no changes in the membrane bulk. Surface modification opens up wide opportunities for the manufacture of novel membranes based on the commercially available ones. Since the cost of surface modification is rather low (due to a small amount of added matter), this method can be of a high practical interest.

It is known that any geometric heterogeneity of the IEM surface leads to a significant increase in the mass transfer rate [5,8,10,24,25]. One of the reasons for this effect is the tangential component of the electric field, which appears in the case of the presence of geometric inhomogeneities on the surface (“hills” and/or “valleys”). The tangential component acts on the space charge at the membrane surface and facilitates the development of EC. Consequently, the formation of a relief on the membrane surface will contribute to an increase in EC intensity. One of the promising methods of surface microstructuring is profiling of IEM [26,27,28,29]. Many studies [25,30,31,32] show that electroconvective vortices—that can significantly increase mass transfer rate—should have a size close to the thickness of the diffusion boundary layer (DBL). For the formation of such vortices, the size of the inhomogeneities on the membrane surface should also be comparable with the DBL thickness.

Regarding the IEM surface charge, as shown in [23], the effect of this property on the mass transfer rate is manifested to the greatest extent at current densities less than or equal to the limiting value. Indeed, it is known that the velocity of fluid electroosmotic slip increases with increasing zeta potential (and, hence, charge) of the membrane surface [33,34]. At underlimiting currents and low potential drops (PD), EC develops by the mechanism of equilibrium electroconvection [34,35], electroosmosis of the first kind [36,37], and the membrane surface charge—determining the rate of electroosmosis—thereby plays a key role in the development of EC [23].

In this paper, we pay more attention to electrical heterogeneity, since obtaining membranes with an electrically heterogeneous surface seems the simplest way of their modification, which leads, however, to a significant improvement in their performance.

In a number of theoretical and experimental studies [38,39,40,41], it was shown that the presence of electrical inhomogeneity on the IEM surface leads to an increase in the intensity of EC. At the same time, the electrical inhomogeneity of the surface also causes negative effects, namely, an increase in the concentration polarization of the solution near the conducting regions of the surface. This phenomenon occurs due to the “funnel effect” [42]. This effect is manifested in a crowding of the electric current lines in the solution near the conductive surface areas. At the same average current density, the accumulation of the electric current lines, i.e., a higher local current density across the conductive areas, leads to a greater decrease in local concentration near the conductive regions in comparison to the case of homogeneous surface. A greater concentration polarization results in a higher voltage across an electrically heterogeneous membrane under the same average current density and under conditions when ion transport occurs by electrodiffusion. At the same time, the “funnel effect” determines the appearance of a tangential electric force that contributes to the development of EC [43,44]. Therefore, a trade-off between the negative and positive manifestations of the funnel effect should exist. It was established, theoretically [38,39], that the appearance of nonconductive regions, which shield a small fraction of the membrane surface, leads not only to intensification of the EC, but also to an increase in the total mass transfer. However, too much shielded surface results in the reduction of mass transfer compared to the homogeneous membrane. Nebavskaya et al. [40] modified homogeneous Neosepta AMX-Sb membranes by deposition of nonconductive strips (parallel to each other) of styrene polyacrylate on their surface. A 2D laser printer was used for the deposition of the strips. It was shown that the limiting current density depends on the fractions of the conducting and nonconducting surfaces. The maximum value of the limiting current density was observed for membranes with a fraction of a nonconducting surface close to 10%.

Another way to change the properties of the membrane surface, which provides the intensification of electroconvection, is the hydrophobization of the membrane surface. The relative hydrophobicity of the membrane surface facilitates the slip of the liquid and leads to an increase in its velocity in the tangential direction near the surface. In theoretical studies [45,46], it was shown that an increase in the hydrophobicity of the surface leads to an increase in the overlimiting mass transfer due to the intensification of EC. To increase the degree of hydrophobicity, thin films of more hydrophobic ion exchange [18] or spots of inert material [21] are deposited on the surface of commercial membranes. As was found in [21], hydrophobic spots of a fluoropolymer, which screen 8% or 12% of the Neosepta AMX anion-exchange membrane surface, allowed increasing the overlimiting current density by about 1.5 times. In addition, the modified membranes showed a lower rate of H^+^ and OH^–^ ion generation than in the case of the pristine membrane.

Despite the fact that some aspects of the effect of electrical inhomogeneity parameters (mainly, the fraction of the screened surface) on the mass transfer rate were studied [21,39], there has not been any real optimization of the surface parameters. Note, that influence of the size of nonconducting regions and the distance between them were considered only in [40]. However, only one type of surface geometry was studied in this paper: alternating conductive and nonconductive strips. Moreover, the nonconductive strips formed protrusions above the conducting membrane surface. Such heterogeneity of the surface geometry did not allow one to clearly distinguish the effect of electrical inhomogeneity. In this paper, we study the effect of the shape and the size of poorly conducting spots as well as the distance between them on the mass transfer rate under conditions where the surface of the modified membrane remains flat. For the first time, we make an attempt to find the optimal parameters of the surface electrical heterogeneity when considering 13 different membrane samples. We carry out this study on a wider range of screened surface fraction values than in [21,40] when applying round, square, and rectangular low conductive spots. The use of stencils made it possible to specify the geometry of the spots with a high accuracy, in contrast to [21], in which the nonconducting material was randomly deposited on the membrane surface.

## 2. Results and Discussion

### 2.1. Membrane Surface Characterization

The characteristics of the commercial and modified membranes are given in Table 1. The latter are obtained by electrospray deposition method, whereby a solution of fluoropolymer Fluoroplast-42 (or F-42) (produced by Plastpolymer, Russia) in an organic solvent (e.g., acetone) is deposited on the surface of a Neosepta AMX or AMX-Sb membrane. The details of the deposition method are given below.

**Figure 1:**
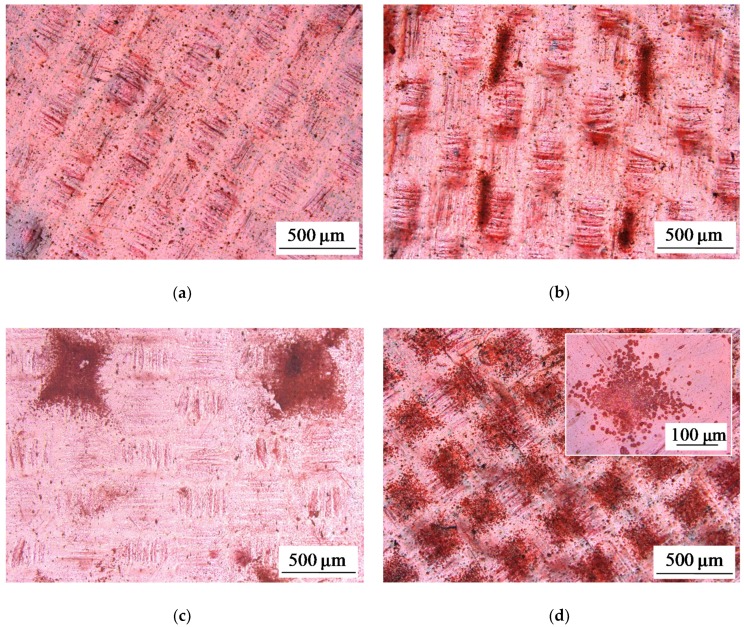
Optical images of the surface of swollen membrane samples: the pristine AMX-Sb membrane (**a**), and the modified AMX-Sb_1_ (**b**), AMX-Sb_2_ (**c**), and AMX-Sb_5_ (**d**) membranes with relatively big spots of fluoropolymer material.

**Figure 2:**
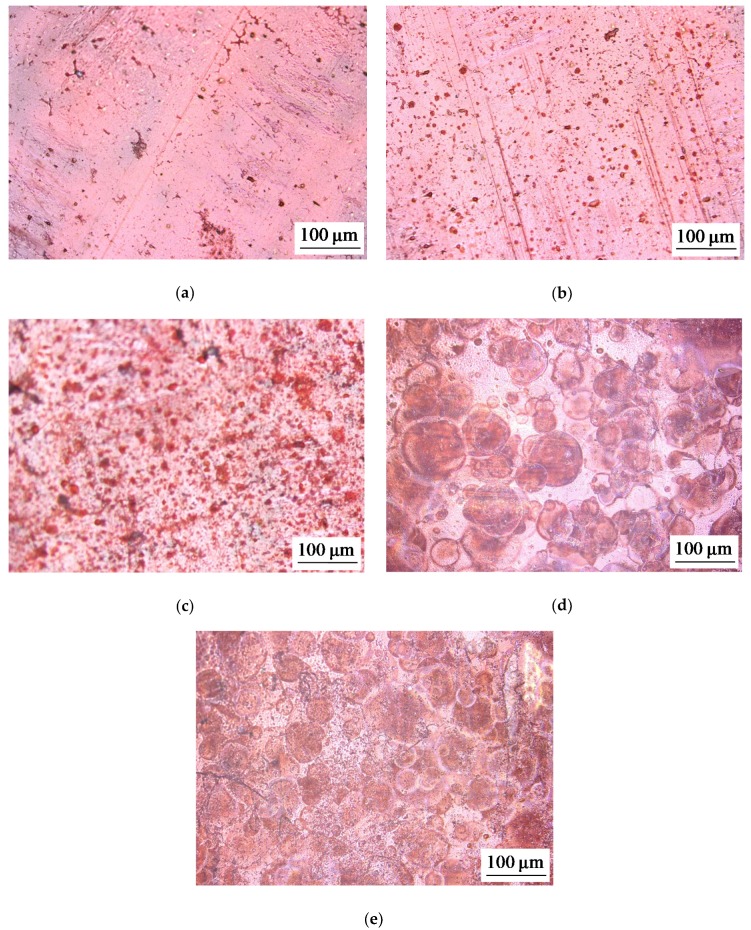
Optical images of the surface of swollen membrane samples: the pristine AMX-Sb membrane (**a**), and the modified AMX-Sb_3_ (**b**), AMX-Sb_4_ (**c**), AMX-Sb_6_ (**d**), and AMX-Sb_7_ (**e**) membranes with relatively small spots of fluoropolymer material.

**Figure 3:**
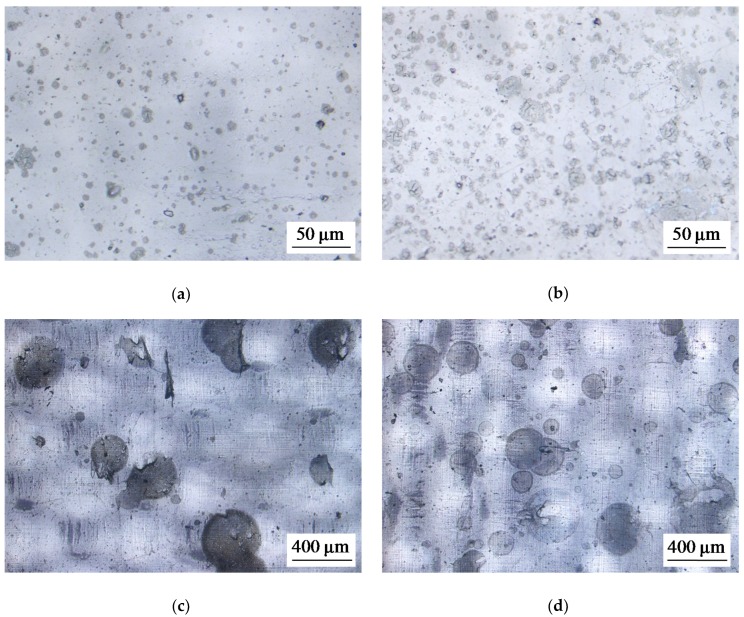
Optical images of the surface of swollen membrane samples: the modified AMX_1_ (**a**), AMX_4_ (**b**), AMX_2_ (**c**), and AMX_3_ (**d**) membranes.

Some of optical images of studied membranes are shown in Figure 1, Figure 2 and Figure 3.

**Figure 4:**
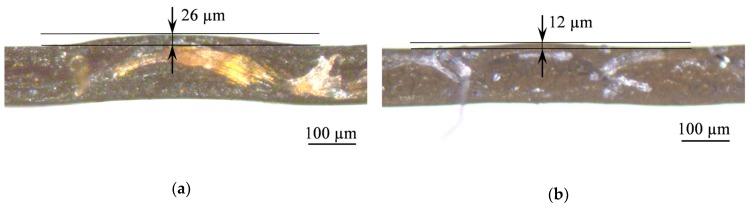
Optical images of cross sections of dry membranes AMX-Sb (**a**) and AMX (**b**). The black arrows show the distance between the black lines indicating the height of the undulation.

The optical images of the cross sections of the studied membranes (Figure 4) show that the AMX-Sb membrane is characterized by a larger height of the undulation, which is determined by the distance from the top of the “hill” to the base of the “valley” on the membrane surface compared to the AMX membrane; this fact has already been noted in [47].

**Figure 5:**
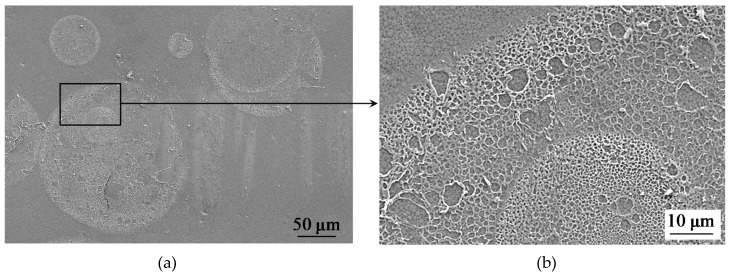
SEM image of the surface of the modified AMX_3_ membrane; (**b**) is the magnification of the region within the black rectangle shown in (**a**).

As the SEM images (Figure 5) demonstrate, the fluoropolymer material deposited on the membrane surface contains pores with the diameter of the order of 1 µm, which could be permeable to both water and ions. Apparently, after deposition of the fluoropolymer–acetone solution, pores/voids are formed in the film upon evaporation of the solvent. Thus, the membrane surface under the modifying film is not fully screened, and it can be expected that the fluoropolymer film is low conductive.

Optical images also show that the relatively large areas perceived as “solid spots” on a scale of 500 μm are, in fact, agglomerates of smaller “spots” with gaps between them. A greater resolution of Figure 1d shows (the insert in Figure 1d) that, in reality, only about 64% of a square seen as a “solid spot” is shielded by the fluoropolymer, while 36% of this square are the gaps that are not screened for the current flow.

**Figure 6:**
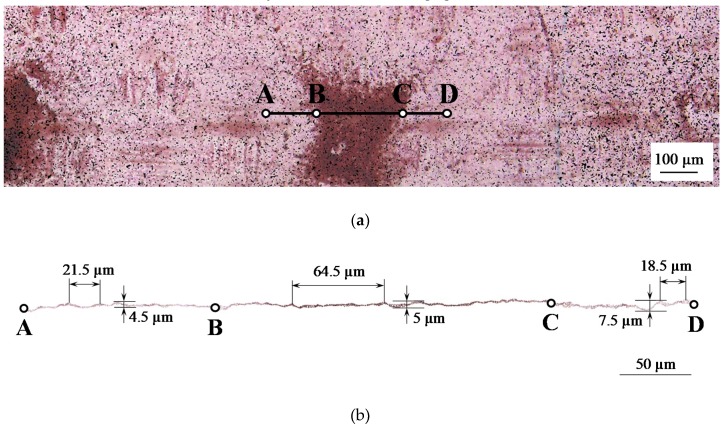
Image of the modified surface with reference points (**a**) and surface profile (**b**) of the AMX-Sb_2_ membrane. Points A and D limit the considered segment of the membrane surface; B and C refer to the boundaries of the fluoropolymer spot.

Figure 6 shows the results of the treatment (using a method described in Section 3. Materials and Methods) of the optical images of the surface of a modified AMX-Sb_2_ membrane. It can be seen that with comparable values of the undulation height (about 5 microns on the spot surface and 4.5–7.5 microns on the pristine surface), the distance between the tops of the “hills” on the spot surface exceeds this value for the pristine surface by three times.

In other words, the surface coated with the spots of the fluoropolymer is smoother than the non-modified surface (Figure 6b). One may conceive that a fluoropolymer solution deposited on the membrane fills the “valleys” on its surface without increasing its thickness. In general, the changes in membrane surface relief caused by modification are negligible.

### 2.2. Conductivity and Contact Angle

**Figure 7:**
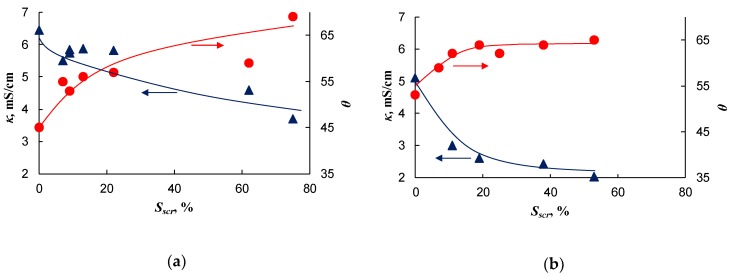
Dependence of the membrane conductivity, *κ*, measured in a 0.5 M NaCl, and the contact angle, *θ*, on the fraction of the screened surface, *S_scr_*. The results of measurements of two series of the modified membranes based on the AMX-Sb (**a**) and AMX (**b**) membranes. The lines are drawn to guide the eye.

The spots of fluoropolymer obtained by electrospinning have a relatively hydrophobic surface. This follows from the fact that with increasing fraction of the screened surface, the contact angle of the membrane surface, *θ*, increases (Figure 7). This property is not surprising since the F-42 fluoropolymer used in this study (see Section 3.1 for details) as modifier has a contact angle equal to 95°. However, with an increasing degree of surface screening, *S_scr_*, the contact angle first rapidly increases, but when *S_scr_* approaches 80%, the rate of increase becomes rather low. However, the value of contact angle remains close to 65°, that is far from 95°. The membrane conductivity, κ, shows a similar behavior. It first rapidly decreases with increasing *S_scr_* (Figure 7), but at relatively high *S_scr_*, the conductivity does not tend to zero, while the F-42 fluoropolymer has a high electrical resistivity equal to 10^9^–10^11^ Ohm·m [48]. The reasons for the observed shape of the contact angle and the conductivity dependence on *S_scr_* are, apparently, due to the fact that the fluoropolymer spots contain pores as was described above (Figure 5). These pores are accessible to the solution with which the membrane is in contact. Thus, even when the entire membrane surface is covered with the fluoropolymer, a part of surface remains open for the ion transfer.

### 2.3. Impact of the Membrane Modification on the Electrochemical Properties

The formation of electrical heterogeneity on the membrane surface is confirmed by the chronopotentiometric data. It is known that in the case of electrically and geometrically homogeneous IEM, there is a transition time, *τ*_Sand_, at currents exceeding the limiting value. The value of *τ*_Sand_ can be calculated from Sand theory [49,50,51] (see Supplementary Material). However, if the IEM surface is electrically inhomogeneous then, as shown in [52], two transition times can occur in a membrane system, even when a binary electrolyte is used as the bathing solution. The occurrence of the first transition time, *τ*_1_, (less than *τ*_Sand_) is due to the fact that the electrolyte concentration near the surface of the conducting regions reaches a certain critical value at which local EC vortices of relatively small size are formed near the boundaries between the conductive and nonconductive surface regions. The second transition time, *τ*_2_, (close to *τ*_Sand_) occurs when the electrolyte concentration becomes low over the entire surface of the membrane, including nonconductive regions. In this case, significantly larger EC vortices arise, causing more intensive mixing of the solution. These vortices significantly reduce the rate of increase (in time) of the PD and lead to the attainment of a stationary state in the system.

**Figure 8:**
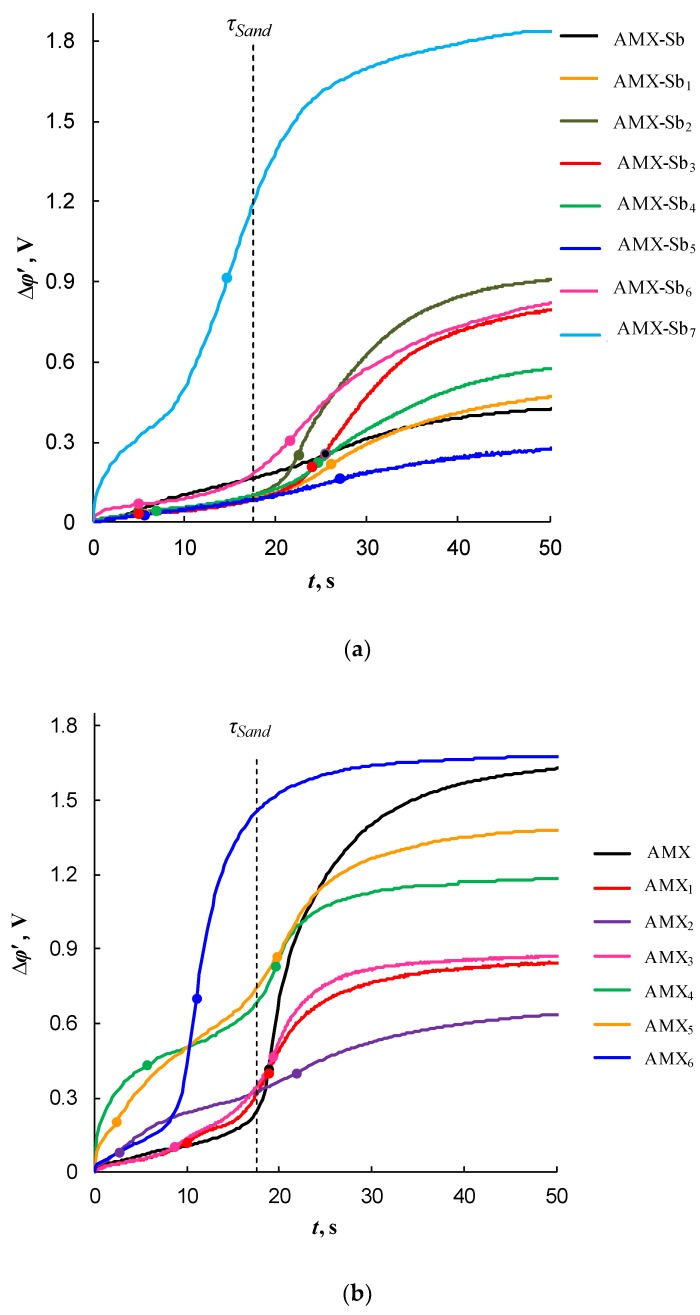
Chronopotentiograms in the cases of pristine and modified AMX-Sb (**a**), and AMX (**b**) membranes measured at $i=1.4\;i_{lim}^{th}$. The circles show the inflection points related to *τ*_1_ and *τ*_2_; the vertical dashed line shows *τ*_Sand_ (Supplementary material).

Figure 8 shows the chronopotentiograms (ChP) of pristine and modified AMX-Sb (a) and AMX (b) membranes measured at current density $i=1.4\;i_{lim}^{th}$, where $i_{lim}^{th}$ is the theoretical value of the limiting current density. The latter is calculated using the Lévêque equation [53] (given in Supplementary Material); in the conditions of our experiments, $i_{lim}^{th}$= 2.8 mA cm^−2^. When plotting ChP, the reduced potential drop (Δ*φ’*) [54] is used. Δ*φ’* is defined as the difference between the total PD, Δ*φ*, and the ohmic PD, Δ*φ*_ohm_, occurring over the unpolarized membrane system just after switching-on the current, the exact definition of Δ*φ’* is given in Supplementary Material.

The appearance of two transition times on the chronopotentiograms of the modified membranes indirectly indicates the electrical heterogeneity of their surface. In addition, as follows from [52], the value of *τ*_2_ can characterize the rate of EC near the membrane surface. As indicated above, EC vortices deliver a fresh solution to the membrane surface and, thereby, delay the increase in PD and the attainment of the stationary state. The more intensive the EC, the greater the experimentally observed value of *τ*_2_ compared to *τ*_Sand_, and the smaller the value of PD in the stationary state. As can be seen from Figure 8, the highest values of *τ*_2_ are observed on the curves characterized by the smallest values of Δ*φ’* is in the case of AMX-Sb_5_ (the AMX-Sb-based series) and AMX_2_ (the AMX-based series) membranes.

**Figure 9:**
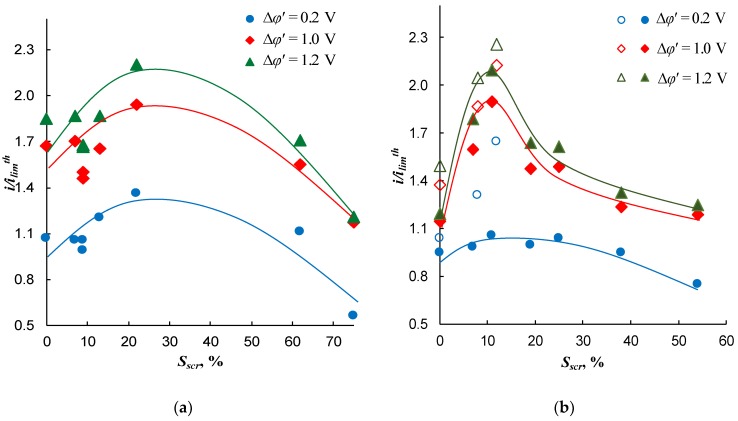
Dependence of the $i/i_{lim}^{th}$ ratio on the fraction of the screened surface for the pristine and modified AMX-Sb (**a**) and AMX (**b**) membranes at the fixed values of the reduced potential drop, Δ*φ’*: 0.2 V (circles), 1.0 V (diamonds), and 1.2 V (triangles). The filled symbols refer to our measurements; the open symbols refer to the results reported in [21]. Lines are drawn to guide the eye.

Figure 9 shows the current density as a function of the fraction of the screened surface area at a fixed reduced PD Δ*φ’* (0.2, 1.0, and 1.2 V). In all cases, this function passes through a maximum, which is in the range between 10% and 20% of the screened surface area.

As can be seen from Figure 9, the greater Δ*φ’*, the greater the current density and the more pronounced the maximum. The presence of a maximum in this dependence is consistent with theoretical estimates in [38,39]. For all considered values of Δ*φ’* , in the case of modified membranes based on AMX-Sb, the maximum value of $i/i_{lim}^{th}$ corresponds to *S_scr_* ≈ 20% (Figure 9a); the best results are shown by the AMX-Sb_5_ membrane (Table 1). As for the AMX-based membranes, the maximum increase in current is achieved in the case of AMX_2_ membrane with *S_scr_* = 11% (Figure 9b). Note that these two membranes were also selected as the best, if based on the chronopotentiometric measurements.

Note also that the larger height of undulation of the AMX-Sb membrane (Figure 4) results in a higher limiting and overlimiting current density of this membrane compared to the AMX membrane. Apparently, this is due to better conditions for the development of electroconvection, as was theoretically established by Rubinstein et al. [32] and other authors [43,55]. In the case of AMX, the limiting and overlimiting current density at a fixed voltage is lower compared to the AMX-Sb membrane. However, the increase in the current density after modification is higher than in the case of the modified membranes based on the AMX-Sb membrane. The best membranes in both series (the AMX-Sb_5_ and AMX_2_ membranes) show close highest values of the $i/i_{lim}^{th}$ ratio, which are slightly below 2.3.

The range of the *S_scr_* values corresponding to the maximum of $i/i_{lim}^{th}$ is close to the theoretical results found by Zabolotsky et al. [39]. According to these estimates, the optimal surface fraction occupied by nonconductive regions is about 10%. The same value of *S_scr_* for the best electrically heterogeneous membranes was also experimentally determined by Nebavskaya et al. [40]. Note that the theoretical optimum value of *S_scr_* estimated by Davidson et al. [38] was approximately equal to 50%. Apparently, the deviation between the theoretical results of Zabolotsky et al. [39] and Davidson et al. [38] was due to the fact that in the model of Davidson the forced fluid flow was assumed to be zero, while this flow (actually occurring under the experimental conditions) was taken into account by Zabolotsky et al.

**Figure 10:**
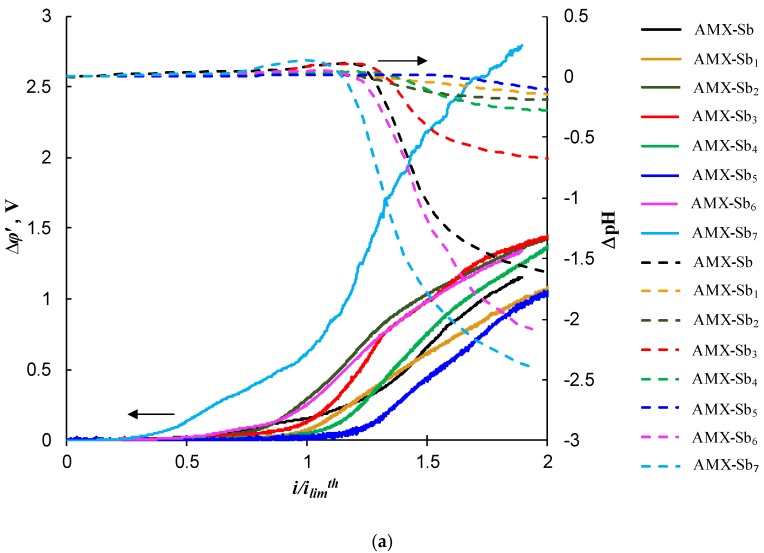

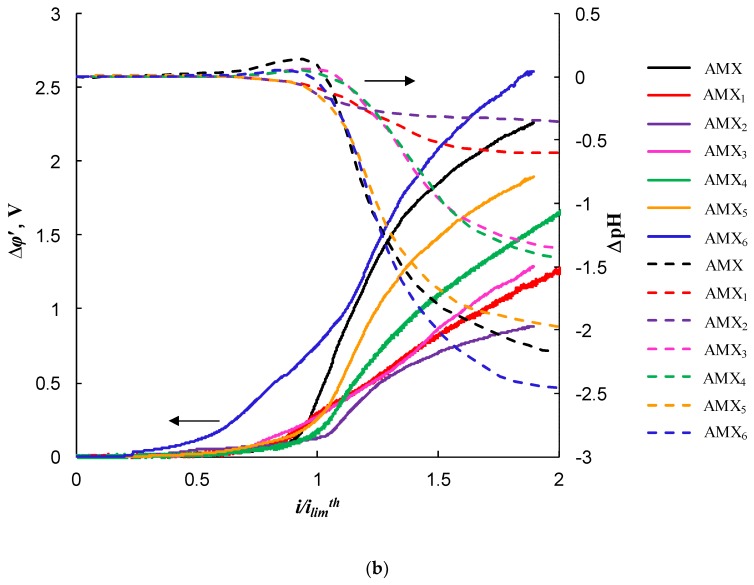
Current–voltage characteristics (solid lines) and the difference in pH between the outlet and inlet solution passing through the desalination compartment of the electrodialysis cell (dashed lines) of the pristine and modified AMX-Sb (**a**) and AMX (**b**) membranes

Figure 10 shows the Δ*φ’* and ΔpH values as functions of the $i/i_{lim}^{th}$ ratio. The value of ΔpH = pH*_out_*–pH*_in_* is determined as the difference in the pH between the outlet and inlet solution passing through the desalination compartment (DC) of the electrodialysis cell. When the H^+^ and OH^−^ ions are formed at the cation-exchange membrane (CEM), the H^+^ ions leave the reaction zone and pass across the CEM into the neighboring compartment; the OH^−^ ions get in the DC. The OH^−^ ions formed at the anion-exchange membrane (AEM) go across the AEM into the concentration compartment, while the H^+^ ions move towards the bulk of DC [5]. Thus, ΔpH depends on the difference between the H^+^ and OH^–^ ion fluxes directed from the depleted surfaces of the CEM and AEM into the DC. If the pH decreases when the solution pass through the DC, it testifies that the water splitting rate is higher at the AEM. Otherwise, the water splitting rate is higher at the CEM. The same auxiliary CEM is used in all the experiments. This membrane forms the DC together with the studied one. In this regard, one can judge the rate of H^+^ and OH^−^ ions generation on the studied AEMs by the value of ΔpH.

As can be seen from Figure 10a, the smaller Δ*φ’* for a given $i/i_{lim}^{th}$ ratio, the lesser the pH changes as the solution passes through the DC. As explained above, the smaller the absolute value of ΔpH at a fixed value of Δ*φ’*, the lower the water splitting rate at the AEM. Conversely, Figure 10a shows that the greater Δ*φ’* at a given $i/i_{lim}^{th}$, the stronger the acidification of the solution when passing through the DC and, hence, the stronger the water splitting at the AEM. This correlation is explained by the fact that intensive electroconvective mixing of the solution near the membrane surface increases the rate of effective mass transfer. In addition, this mixing causes an increase in the concentration of salt ions in the surface layer, which leads to a decrease in the rate of H^+^ and OH^–^ ion generation. For the AMX membranes (pristine and modified), similar results were obtained, as shown in Figure 10b.

The following discussion focuses on the AMX-Sb membrane and samples obtained by its modification. The reasons behind the differences in the electrochemical characteristics of the pristine and modified samples are similar in the cases of both the AMX and AMX-Sb series.

The membranes having the surface screened by more than 50% (AMX-Sb_6_, AMX-Sb_7_, AMX_6_) are characterized by a high rate of H^+^ and OH^–^ ion generation (Figure 10) and a rather low value of $i/i_{lim}^{th}$ ratio at a fixed Δ*φ’*. This indicates a low effective mass transfer, lower than in the case of the pristine membrane and in the case of membranes with a small portion of the screened surface.

**Figure 11:**
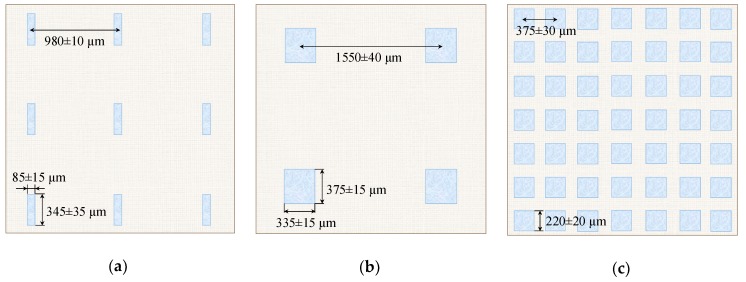
Schematic view of the surface of the AMX-Sb_1_ (**a**), AMX-Sb_2_ (**b**), and AMX-Sb_5_ (**c**) membranes. The same scale is used for all membranes.

**Figure 12:**
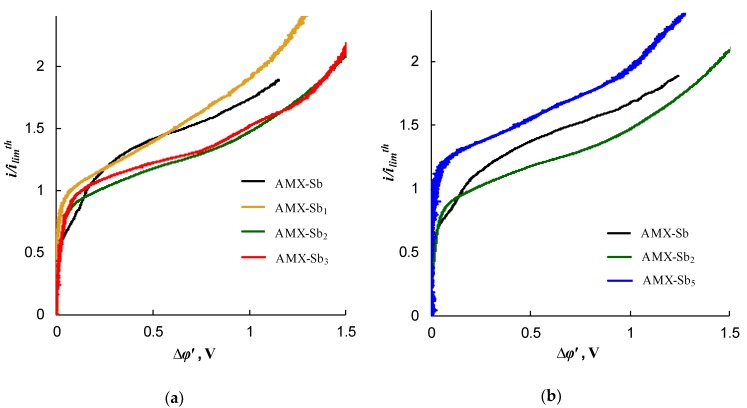
Current–voltage characteristics of the pristine AMX-Sb membrane and different modified membranes: (**a**) AMX-Sb_1_, AMX-Sb_2_, and AMX-Sb_3_ membranes having nearly the same *S_scr_*≈ 10%; (**b**) AMX-Sb_2_ and AMX-Sb_5_ membranes having nearly the same size of spots and different values of *S_scr_*, for the AMX-Sb_5_ membrane *S_scr_* ≈ 22%.

In order to understand what sizes of poorly conducting spots and the distances between them are significant, we considered three modified membranes (AMX-Sb_1_, AMX-Sb_2_, and AMX-Sb_3_) having approximately the same fractions of the screened surface, which are in the range 7%–9%. Figure 11a,b shows a scheme of the spots’ distribution on the surface of the AMX-Sb_1_ and AMX-Sb_2_ membranes, respectively. Figure 12a gives the current–voltage characteristics (CVC) for these three membranes together with the pristine AMX-Sb membrane. As can be seen from Figure 12a, the CVC of AMX-Sb_1_ is almost the same as that of the AMX-Sb membrane, while the I–V curves of AMX-Sb_2_ and AMX-Sb_3_ lie noticeably lower. Apparently, the obtained results can be explained by the peculiarities of the EC vortices’ development near the membrane surface. We proceed from the fact that the presence of boundaries between the well and poorly conducting surface regions causes the occurrence of a tangential component of the electric force. The action of this force on the space charge region near the conducting surface causes the formation of an EC vortex. As shown in the works of Rubinstein [32] and other authors [19,45,55], EC vortices are formed both near a heterogeneous and near a smooth electrically homogeneous surface. However, the presence of electrical or geometric heterogeneity causes the occurrence of relatively big vortices [31,32,56] at lower values of PD (the early onset of EC instability).

**Figure 13:**
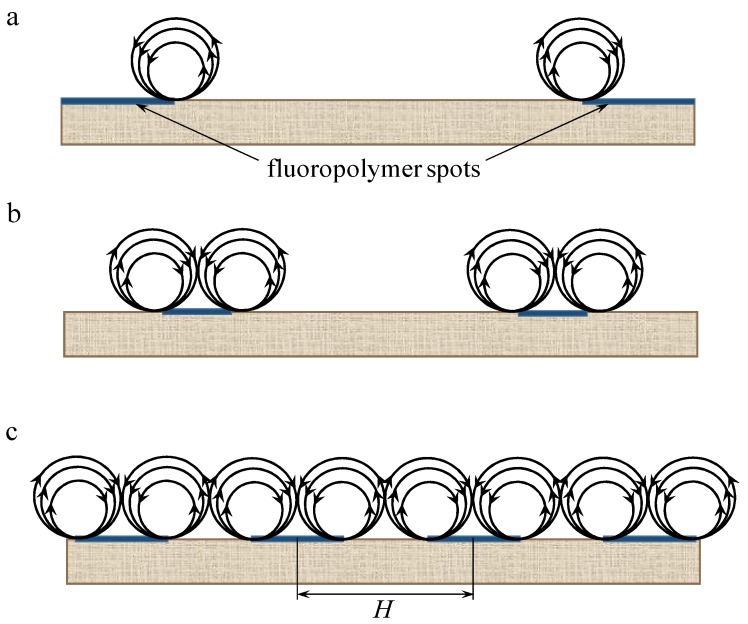
Scheme of the distribution of electroconvective vortices at the surface of AMX-Sb_2_ (**a**), AMX-Sb_1_ (**b**), and AMX-Sb_5_ (**c**). *H* is the minimum distance between the repeating elements of the surface heterogeneity.

It follows from theoretical considerations [32,55] that the shape of EC vortices is close to a circle. When there is a forced flow, it affects the vortices and determines their maximum size, which can hardly be greater than the diffusion layer thickness; the latter is about 250 μm in the conditions of the ED cell design and flow rates used, see Section 3. On the other hand, as the vortices arise at the boundaries of well and poorly conducting surface regions, their maximum diameter cannot be more than half the distance *H* between the repeating elements of the surface heterogeneity (Figure 13c).

Apparently, the size of the fluoropolymer spots forming electrical heterogeneity regions on the surface of the AMX-Sb_3_ membrane is too small (close to 5 μm) to affect significantly the intensity of EC; these spots differ only little in size from the defects present on the surface of the pristine AMX-Sb membrane (compare Figure 2a,b). However, these spots screen a part of the AMX-Sb_3_ surface, and this causes a decrease in the current density at a given PD due to the development of the “funnel effect”. As for the AMX-Sb_2_ membrane, there are relatively large fluoropolymer spots on its surface. These regions are located far from each other (Figure 1c and Figure 11b). This should lead to the development of larger vortices. Nevertheless, due to the large distance between the spots, the number of vortices per surface unit is quite small (Figure 13a). This is the reason for the predominance of the concentration polarization factor over the EC intensification factor for this membrane, which explains the fact that the CVC of the AMX-Sb_2_ membrane lies lower than that of the pristine membrane.

In the case of the AMX-Sb_1_ membrane (Figure 1b and Figure 11a), the size of the spots is smaller than in the case of the AMX-Sb_2_ membrane. However, it can be expected that since the size of the spots on the AMX-Sb_1_ membrane is close to the diffusion layer thickness, the size of the vortices near this membrane would be approximately the same as near the AMX-Sb_2_ membrane. Nevertheless, the distance between the vortices is smaller than in the case of AMX-Sb_2_ (Figure 13b), which leads to an increasing number of vortices per unit surface area. More vortices at the surface of the AMX-Sb_1_ membrane result in increasing mass transfer and the fact that the current density across this membrane is higher than that across the pristine AMX-Sb membrane (Figure 12a).

The comparison of the CVCs of AMX-Sb_1_ and AMX-Sb membranes shows that the current density across the modified membrane is higher than that across the pristine membrane at low reduced potential drops (<0.2 V) and in the range of high PD (>0.5 V). The better performance of the modified membrane at low PD is apparently explained by earlier upset of electroconvection developing by the mechanism of equilibrium electroosmosis of the first kind [34,35,36,37]. The presence of boundaries of well and poorly conducting regions facilitates electroosmotic flow, which is enhanced by hydrophobic surface of the fluoropolymer spots. The undulated surface of AMX-Sb membrane contributes to the emergence of this type of electroconvection. At higher PD, electroconvection becomes unstable; evidently the presence of hydrophobic spots contributes also to a more intensive development of this non-equilibrium kind of electroconvection [55,56].

The location of the conductive and screened surface regions, as well as the vortices formed at their boundaries, are shown schematically for the AMX-Sb_2_ and AMX-Sb_1_ membranes in Figure 13a,b, respectively. A higher density of vortices near the AMX-Sb_1_ membranes apparently causes higher mass transfer rate than in the case of the AMX-Sb_2_ membrane.

As Figure 12b shows, the overlimiting current density through the AMX-Sb_5_ membrane is essentially higher than through the pristine and the AMX-Sb_2_ membranes. Figure 11c shows the distribution of poorly conducting spots on the surface of the AMX-Sb_5_ membrane, having the screened surface fraction value of 22%, that is, more than twice as much as that of AMX-Sb_2_. Compared to the AMX-Sb_2_ membrane, the spots are only slightly smaller, but they are more densely located. As a result, the size of the vortices near the surface of the AMX-Sb_5_ membrane should not differ much from those formed near the surface of AMX-Sb_2_, but the density of vortices distribution near the AMX-Sb_5_ membrane will be essentially higher (Figure 13a,c). The same can be said when comparing the AMX-Sb_5_ and AMX-Sb_1_ membranes. As a result, the factor of EC intensification becomes dominant, and a significant increase in the mass transfer rate is observed compared to the pristine membrane (Figure 12b).

Approximate evaluations using known sizes of fluoropolymer spots on the surface of the AMX-Sb_5_ membrane show that vortices with a diameter of about 190 μm densely occupy the surface (Figure 13c). An important circumstance is that the diameter of the vortex is close to the thickness of the diffusion layer. As was shown in [30], the vortices of such sizes most effectively mix the solution and reduce the concentration polarization near the membrane surface. The vortices that are close in size to the DBL thickness, *δ*, take the “fresh” solution from the solution bulk and deliver it to the membrane surface. At the same time, they take away the depleted solution from the near-membrane space into the bulk solution. The smaller vortices (as compared to *δ*) stir the solution only near the surface, and the larger ones, though more efficient, require greater energy consumption, since they are more affected by the forced flow.

Let us also note that the vortices formed at the membrane surface are paired under the described conditions: the movement of the fluid is directed from the center of the nonconducting (poorly conducting) regions to the conducting regions; the direction of rotation of the neighboring vortices is the opposite—if one of them rotates clockwise, then the neighboring vortex rotates counterclockwise (Figure 13c) [19,32].

It seems that the degree of hydrophobicity of the polymer spots on the membrane surface is also important. Indeed, according to theoretical [43,45,46] and experimental [18,23] works, the higher the degree of surface hydrophobicity, the easier the fluid slips along the surface. Therefore, it can be expected that the use of a polymer, which is more hydrophobic than the fluoropolymer used in this study (whose contact angle is 95°), could provide a greater mass transfer enhancement than in the actual study. This topic is in the plan of our further work.

## 3. Materials and Methods

### 3.1. Membranes

Commercial Neosepta homogeneous anion-exchange AMX and AMX-Sb membranes manufactured by Astom, Tokyo, Japan, were used as the ion-selective substrate for preparing the samples with different electrical heterogeneity. The pristine membranes contain a poly(styrene-divinylbenzene) copolymer ion-exchange matrix with fixed quaternary ammonium bases as functional groups. Both AMX and AMX-Sb membranes have high permselectivity, electrical conductivity, and mechanical strength.

The membrane modification is carried out using the electrospray deposition method [21,57,58]. For the formation of hydrophobic spots on the membrane surface, a hydrophobic low molecular weight fluoropolymer Fluoroplast-42L (or F-42L) [48] (JSC “Plastpolymer”, Saint Petersburg, Russia), which is a poly(vinylidene fluoride-tetrafluoroethylene) copolymer with structural formula[-CF2-CF2-]n-[-CH2-CF2-]1-n, was selected. The mass fraction of moisture in F-42L is not more than 0.05%; the volume resistivity is 10^9^–0^11^ Ohm·m; the density is 1900–2000 kg m^−3^; the ratio of the viscosity of the 0.01 g cm^−3^ F-42L solution in acetone, and that of the pure acetone is 2.5–3.7 [48]. A 0.3% solution of the F-42L in acetone was prepared.

The membranes before modification were stored in a 0.02 M NaCl solution, then they were dried to an air-dry state.

**Figure 14:**
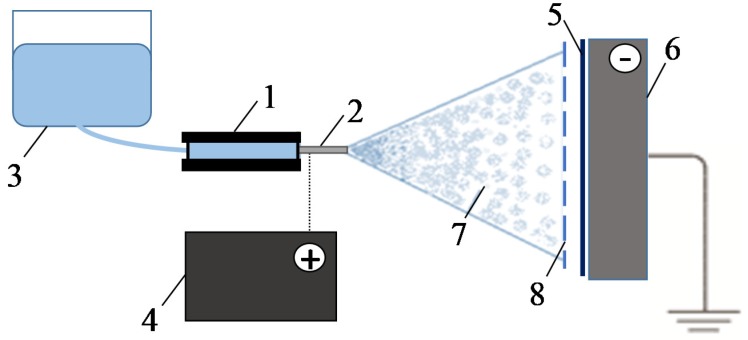
Principal scheme of the setup for the membrane surface modification: 1—high-precision syringe pump, 2—metal needle, 3—polymer solution, 4—high voltage source, 5—modified membrane, 6—cathode, 7—stream of drops of polymer solution, 8—template.

The schematic diagram of the setup, which was used to modify the AEMs surface, is shown in Figure 14. A detailed description of the setup and method of coating the surface of the AEM membranes with fluoropolymer spots is given in [59]. The setup consists of three main parts: electrical, mechanical, and hydraulic. The electrical part includes a high voltage source and polarizing electrodes. The mechanical part allows one to move the sprayer in two axes, ensuring uniform application of fluoropolymer. The hydraulic part consists of a high-precision syringe pump (1) and a metal needle (2) to spray the polymer solution (3) with a given rate and given size of the drops. A positive electric potential is applied by a voltage source (4) to the needle (2). The membrane (5) is located between the positively charged needle (2) and a flat grounded cathode (6). Under the action of an electric field, the positively charged microdroplets of polymer solution (7) move towards the cathode. The use of nylon template (8) allows obtaining the spots of different shape on the membrane surface.

Membrane modification is carried out in the air at room temperature (from 20 to 25 °C). The concentration of the fluoropolymer in acetone, duration of spraying (from 3 to 120 s), the needle movement speed along the membrane surface (from 0.25 to 2 cm s^−1^), the distance from the tip of the needle to membrane surface (from 3 to 7 cm), and the tension value (from 2 to 7 kV cm^−1^) are the parameters to be optimized. It was experimentally established that, in addition to spots, fluoropolymer fibers appear on the surface of the membranes if the concentration of fluoropolymer in acetone exceeds 0.3%. The duration of spraying and the voltage value are the parameters to be optimized. If the voltage is too high, the flight speed of the polymer solution droplets is too high and the droplet size is small, so that the solvent evaporates before reaching the membrane surface. With insufficient voltage, the droplets as well as the spray angle becomes too large. The greater the distance from the tip of the needle to the membrane, the higher the spray angle and, accordingly, the less the number of spots per unit membrane surface area. The optimum values of voltage and spraying time were experimentally established.

The pristine and modified membranes underwent conventional salt pretreatment [58] before the measurements.

It is known that the exposure of the F-42 polymer or a similar polymer to a strong electric field leads to the formation of chemical bonds with oxygen [60,61,62], in particular, carboxyl groups. The result of such chemical transformations is in the acquisition by F-42L of a negative electric charge. In our case, these groups provide good adhesion of F-42L with the positively charged amino groups on the AEMs surface. Apparently, for this reason the modified samples showed stable characteristics during the study (more than 100 h).

### 3.2. Methods

#### 3.2.1. Surface Visualization

The images of swollen membranes were obtained using an optical metallographic microscope SOPTOP CX40M (Ningbo Sunny Instruments Co., Ltd., Yuyao, China). The surface of the membranes is also studied using a Hitachi TM3000 scanning electron microscope (SEM) (Hitachi High-Technologies Corp., Tokyo, Japan).

The fraction of the surface area covered with fluoropolymer spots (fraction of the screened surface, *S_scr_*) for the modified membranes is determined by treating the optical images using the ToupView 3.7 software (Hangzhou ToupTek Photonics Co., Ltd., Hangzhou, China); the details of a similar method are described in [63].

**Figure 15:**
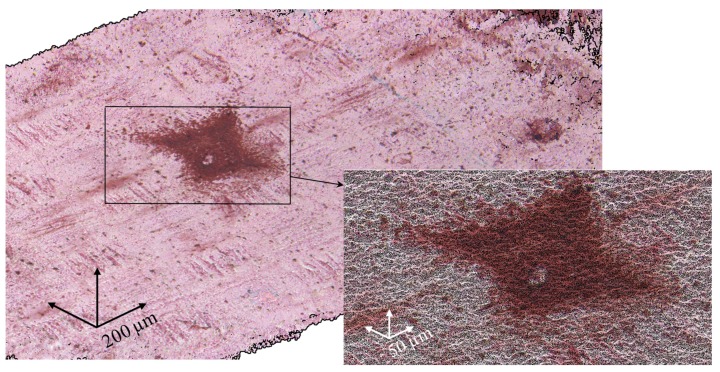
3D image of the AMX-Sb_2_ membrane in the form of photogrammetric point cloud.

3D images of the ion-exchange membranes surface as well as a detailed description of their relief were carried out using geographic information software QGIS 3.7. For this purpose, a series of micrographs taken in a certain way was treated. The model of the surface was obtained in the form of a photogrammetric point cloud. Figure 15 shows an image of the AMX-Sb_2_ ion-exchange membrane surface, which was formed on the basis of 37 micrographs.

Based on the data obtained, a number of surface profiles were plotted. The height of undulation as well as the distances between the peaks of the “hills” on the membrane surface were measured using the Saga 2.0.5 software (SAGA User Group Association, Hamburg, Germany). The darker area on the AMX-Sb_2_ membrane, corresponding to the fluoropolymer-coated region, has different morphometric properties than the lighter area not coated with the modifier (Figure 15).

#### 3.2.2. Voltammetry and Chronopotentiometry

**Figure 16:**
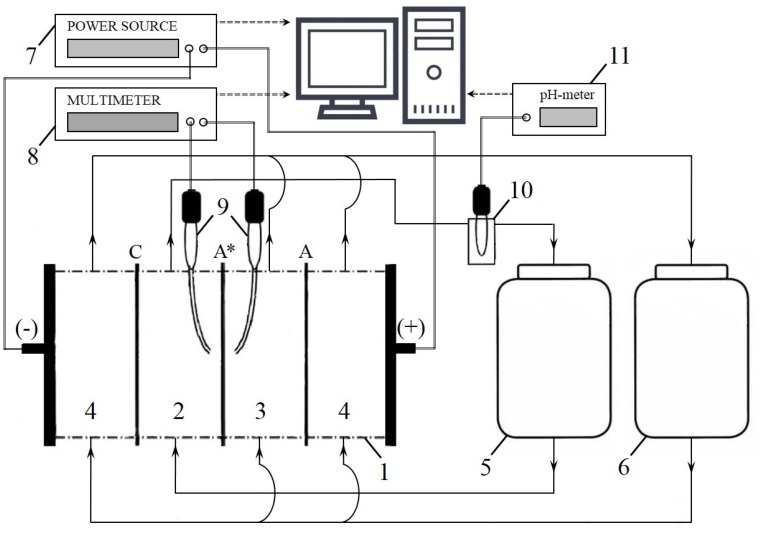
Principal scheme of the experimental setup: electrodialysis cell (1) consisting of desalination (2), concentration (3), and two electrode compartments (4); tanks with solutions (5, 6); programmable power source Keithley SourceMeter 2400 (7); multimeter Keithley 2010 (8); Luggin’s capillaries, connected with silver chloride electrodes (9); flow pass cell with pH combination electrode (10); pH-meter Expert 001 (11).

The electrochemical characteristics, the current–voltage curves, and chronopotentiograms of the studied membranes were obtained using the experimental setup shown in Figure 16. The setup includes hydraulic and measuring systems, as well as a laboratory four-compartment flow electrodialysis cell (1) consisting of desalination (2), concentration (3), and two electrode compartments (4). The compartments of the cell are formed by the studied anion-exchange membrane (A*) and auxiliary heterogeneous cation-exchange MK-40 (C) and anion-exchange MA-41 (A) membranes. The intermembrane distance is 6.5 mm, the polarized membrane area is 2 × 2 cm^2^. The desalination compartment (DC) is fed with a 0.02 M NaCl solution (the volume of which is 1 dm^3^) from tank (5), the concentration and electrode compartments are supplied with a 0.02 M NaCl solution (the volume of which is 5 dm^3^) from the tank (6). The average linear flow velocity of the solutions in all compartments is equal to 0.38 cm s^−1^. The electrical conductivity and the pH values of the solutions in tanks (5) and (6) were continuously monitored. In preliminary experiments, it was found that due to relatively high volume of desalination and concentration/electrode streams and low flow rate, the changes in electrical conductivity and pH values in both tanks did not exceed 1% during each experimental run. The solutions in the tanks were replaced with fresh ones before each experimental run. The measurements of the pH were carried out in tank (5) and in flow pass cell with pH combination electrode (10) using pH-meter Expert 001 (Econix-Expert, Ltd., Moscow, Russia) (11); in this way, the pH difference between the inlet and outlet DC solutions was determined.

A direct current is supplied to the cell by a power source Keithley SourceMeter 2400 (Keithley Instruments, LLC, Solon, OH, USA) (7). A potential drop across the studied membrane is determined using Luggin’s capillaries, connected with silver chloride electrodes (8) and registered by multimeter Keithley 2010 (Keithley Instruments, LLC, Solon, OH, USA) (8). The experimental procedure is detailed in [63]. The experiments were conducted at 20 °C.

The electrodialysis cell (1) design (in particular, the special input and output devices of the solution) provide a laminar flow of the solution in the intermembrane space [63] (see Supplementary Material). This condition allows theoretical calculation of the limiting current density ($i_{lim}^{th}$) using the Lévêque equation [63] and the diffusion layer effective thickness (*δ*) using the combination of the Lévêque and Peers [64] equations. In the conditions of our experiments, this calculation gives $i_{lim}^{th}$ = 2.8 mA⋅cm^−2^, *δ* = 250 μm.

The contact angles (*θ*) of the swollen membranes under study are measured 20 s after applying a drop of 0.02 M NaCl solution on the membrane surface. The measurement technique is described in detail in [65].

The conductivity of the membranes is measured by the differential method using a clip-type cell [66,67] and a LCR meter RLC AKIP-6104 (JSC “PriST”, Moscow, Russia) at an AC frequency of 1 kHz in a 0.5 M NaCl solution.

The thickness of membranes was measured using a digital micrometer Schut Filetta (Schut Geometrical Metrology, Groningen, Netherlands). The thickness of the pristine AMX-Sb, AMX membranes and modified membranes (on the basis of AMX-Sb and AMX) turned out to be identical within the measurement error (128 ± 5 μm).

## 4. Conclusions

It has been shown that the deposition of relatively hydrophobic poorly conducting fluoropolymer spots on the surface of a homogeneous membrane (at the fractions of the screened surface in the range of 10%–20%) allows increasing mass transfer rate by up to 1.5 times and reducing water splitting rate near the membrane surface. In this case, it is possible to turn the factor of EC intensification into dominant over the factor of increasing concentration polarization (CP). EC is due to the formation of vortices at the boundaries of the well conducting and poorly conducting regions; the increase in CP is due to the “funnel effect” (which is the accumulation of the electric current lines on the conducting regions of the membrane surface).

It was established that the shape of poorly conducting fluoropolymer spots did not have a significant effect, while the size of the screened areas and the distance between them play an important role in the development of EC. If the size of the screened regions is too small (less than 10 μm), then the size of the EC vortices formed at the boundaries of the conducting and poorly conducting sections, is apparently also small, and the factor of EC intensification does not exceed the factor of increase in CP. When the size of nonconducting regions is of a few hundred micrometers (that is comparable with the DBL thickness), the size of the formed EC vortices, apparently, reaches approximately the same values.

However, if the distance between the neighboring screened regions significantly exceeds the DBL thickness, as in the case of the AMX-Sb_2_ membrane, the number of vortices per unit surface of the membrane is too low to provide an increase in mass transfer compared to the pristine membrane. If the screened regions are located at a distance slightly exceeding the DBL thickness then a fairly dense distribution of vortices at the membrane surface can be expected. Indeed, the surface of AMX-Sb_5_ membrane is characterized by such parameters and provides the maximum (among the studied samples) increase in mass transfer in comparison with the pristine membrane.

## Figures and Tables

**Table 1:** Surface characteristics of the membranes under study.

Sample	Spot Shape	Spot Size ^1^, μm	The Distance Between the Centers of the Spots, μm	Fraction of the Screened Surface, *S_scr_*, %	Contact Angle, *θ*, Degrees
AMX-Sb	-	-	-	0	45 ± 2
AMX-Sb_1_	rectangle	85 (±15) × 340 (±35)	980 ± 10	7 ± 1	55 ± 3
AMX-Sb_2_	rectangle	335 (±15) × 375 (±15)	1550 ± 40	9 ± 1	53 ± 1
AMX-Sb_3_	circle	3 ÷ 10	6 ÷ 22	9 ± 1	53 ± 3
AMX-Sb_4_	circle	3 ÷ 15	7 ÷ 21	13 ± 1	56 ± 1
AMX-Sb_5_	square	220 (±20) × 220 (±20)	375 ± 30	22 ± 2	57 ± 2
AMX-Sb_6_	circle	25 ÷ 80	5 ÷ 25	62 ± 3	59 ± 4
AMX-Sb_7_	circle	50 ÷ 135	10 ÷ 30	75 ± 3	69 ± 1
AMX	-	-	-	0	53 ± 1
AMX_1_	circle	3 ÷ 7	6 ÷ 20	7 ± 1	59 ± 1
AMX_2_	circle	60 ÷ 400	40 ÷ 170	11 ± 1	62 ± 2
AMX_3_	circle	20 ÷ 400	10 ÷ 40	19 ± 1	64 ± 2
AMX_4_	circle	1 ÷ 14	4 ÷ 14	25 ± 1	62 ± 3
AMX_5_	circle	4 ÷ 60	20 ÷ 60	38 ± 1	64 ± 3
AMX_6_	circle	30 ÷ 200	10 ÷ 22	53 ± 1	65 ± 2

^1^ In the case of circle spots, their diameter is indicated; in the case of rectangular and square spots, the length and width are indicated.

## Supplementary Materials

Supplementary Materials can be found at https://www.mdpi.com/1422-0067/21/3/973/s1.
