# Supplementary material for: How Electrical Heterogeneity Parameters of Ion-Exchange Membrane Surface Affect the Mass Transfer and Water Splitting Rate in Electrodialysis
Source: Int J Mol Sci. 2020 Feb 1;21(3):973. doi: 10.3390/ijms21030973 (PMC7037469; doi:10.3390/ijms21030973)
Supplement: Supplementary file 1 [file ijms-21-00973-s001.pdf]

Calculations of the limiting current density and the transition time

The electrodialysis cell (1) design (in particular, the special input and output devices of the solution) provide a laminar flow of the solution in the intermembrane space [63]. The laminarity of the hydraulic regime was proved by using CFD simulations and by comparing the experimental limiting current density with the value calculated using the Lévêque equation, which was deduced under the assumption of laminar flow in the intermembrane space [63].

The theoretical limiting current density, $i_{\lim}^{th}$, which is achieved in the absence of coupled effects of concentration polarization (such as electroconvection, gravitational convection, and water splitting) can be calculated by the Lévêque equation [53]:

$$i_{\lim}^{th} = \frac{FDz_1C_1}{h(T_1 - t_1)}\left[\left(1.47\frac{h^2V}{LD}\right)^{1/3} - 0.2\right] \qquad (1)$$

where F is the Faraday constant; D is the electrolyte diffusion coefficient; z_1 and C_1 are the charge and the input molar concentration of the counterion; T_1 and t_1 are the effective transport number of the counterion (Cl^- in the considered cases) in the membrane and its transport number in solution; V is the average linear flow velocity; L is the length of the desalination path; h is the intermembrane distance.

The parameters used in the experiments (at 20 °C) are as follows: D=1.425·10^{-9} m^2/s, T_1 is assumed to be 1, t_1 is 0.605, V is 0.38 cm·s^{-1}, h is 6.5 mm, and L is 2 cm. The value calculated from Eq. (1) for these experimental conditions is 2.8 mA·cm^{-2}.

The limiting current density $i_{\lim}^{th}$ is related to the diffusion layer effective thickness, δ, by the Peers equation [64]:

$$i_{\lim}^{th} = \frac{FDz_1C_1}{\delta(T_1 - t_1)} \qquad (2)$$

A combination of Eqs. (1) and (2) gives δ equal to 250 μm.

To compare chronopotentiograms of different membrane systems, it is convenient to use the reduced potential drop $\Delta\varphi'$ [54] instead of the total potential drop, $\Delta\varphi$:

$$\Delta\varphi' = \Delta\varphi - \Delta\varphi_{ohm} \tag{3}$$

where the ohmic potential drop, $\Delta\varphi_{ohm}$, of the unpolarized membrane system is found by the extrapolation in the $\Delta\varphi - \sqrt{t}$ coordinates to zero time (the time of the current switch-on).

The theoretical transition time, τ_{Sand}, is found using the Sand equation [49]:

$$\tau_{Sand} = \frac{\pi D}{4}\left(\frac{F z_1 C_1}{T_1 - t_1}\right)^2 \frac{1}{i^2} \tag{4}$$

Eq. (4) allows estimating the transition time in the case where the membrane surface if homogeneous and smooth, and electrolyte transfer is governed only by electro-diffusion.

The experimental current-voltage curves are plotted in the coordinates of the ratio of the current density to its theoretical limiting value $i/i_{\lim}^{th}$ versus the reduced potential drop $\Delta\varphi'$. In this case $\Delta\varphi'$ is related to the measured potential drop as

$$\Delta\varphi' = \Delta\varphi - iR_{i=0} \tag{5}$$

where $\Delta\varphi$ is the measured value of potential drop at a current density i and $R_{i=0} = \left(\partial\Delta\varphi / \partial i\right)_{i\to 0}$ is the resistance of the membrane system at $i \to 0$.
